# “Wesley says”: a children’s response inhibition playground training game yields preliminary evidence of transfer effects

**DOI:** 10.3389/fpsyg.2015.00207

**Published:** 2015-02-25

**Authors:** Xin Zhao, Ling Chen, Lily Fu, Joseph H. R. Maes

**Affiliations:** ^1^Behavior Rehabilitation Training Research Institution, School of Psychology, Northwest Normal UniversityLanzhou, China; ^2^Centre for Cognition, Donders Institute for Brain, Cognition and Behavior, Radboud UniversityNijmegen, Netherlands

**Keywords:** response inhibition training, transfer effects, go/no-go task, Stroop color–word interference task, children

## Abstract

Recent studies suggest that the response inhibition ability of children can be modified through training. Based on the notion of embodied cognition, we investigated transfer effects of a 7-day training program using a game named “Wesley says” in 8- to 12-year-old children (*n* = 15). The game consists of providing commands for performing simple body actions, the actual execution of which is conditional upon the preceding verbal expression “Wesley says.” Training effects were assessed with a computer-based visual go/no-go task and the Stroop color–word interference task. Relative to a control group playing other games mainly involving physical exercise (*n* = 15), the trained group showed a performance improvement on the go/no-go task, but not on the Stroop task. These results suggest the potential of an easy-to-use and ecologically valid training game to improve the inhibition capacity of children on related response inhibition tasks but not on tasks measuring other aspects of inhibition, such as interference control.

## INTRODUCTION

Response inhibition refers to the ability to postpone, withhold, or stop inappropriate behavior (Bari and Robbins, 2013). The most commonly used research paradigms of (motor) response inhibition include the anti-saccade task (Munoz and Everling, 2004), the go/no-go task (Trommer et al., 1988), and the Stop-signal task (Logan, 1994). Studies suggest that response inhibition ability as measured with such tasks has predictive value with respect to individual health (Penadés et al., 2007; Spronk et al., 2008), mathematical and reading skills in early childhood (Blair and Razza, 2007), and social functioning (Carlson and Moses, 2001).

In view of the importance of inhibition abilities, recent research has assessed whether these can be improved trough training. Part of this research revealed that, in children, at least some aspects of response inhibition can indeed be modified (e.g., Dowsett and Livesey, 2000; Thorell et al., 2009; Johnstone et al., 2010, 2012; Motes et al., 2014). For example, Dowsett and Livesey (2000) trained 3- to 5-year-old children who largely lacked inhibitory control abilities as measured by a go/no-go task. After training of aspects of executive functioning using a variant of the Stop-signal and Wisconsin Card Sort tasks (presumably training attentional shifting and working memory), the children’s response inhibition ability on the go/no-go task had improved significantly. Johnstone et al. (2010) performed a study with a sample of 7- to12-year-old children with AD/HD who completed a 5-week training program using a go/no-go task. They reported trends for improved performance after training on a related non-trained go/no-go task and significant reductions in symptom frequency as reported by parents and relatives of the children. In addition, Johnstone et al. (2012) examined the effects of a combined working memory and response inhibition (using a go/no-go task) training program on the performance of 7- to 13-year-old children with and without AD/HD on a non-trained go/no-go task, measuring response inhibition, and a Flanker task, measuring interference control (see Section “Discussion” for a more detailed reflection on these types of inhibition). The authors found that after training AD/HD symptoms were near-significantly alleviated and interference control was significantly improved. However, there were no significant effects on response inhibition. Motes et al. (2014) trained 12- to 15-year-old children using a cognitive strategy training program (the Strategic Memory Advanced Reasoning Training program, Gamino et al., 2010). They found that training improved performance on a go/no-go task. Finally, Thorell et al. (2009) trained 4- to 5-year-old children using a program that either targeted working memory or inhibition. The inhibition training improved performance on the trained inhibition tasks, which included measures of response inhibition and interference control. However, none of the training programs improved performance on non-trained inhibition tasks.

These studies implicate mixed results with respect to the potential of response inhibition training to improve inhibition capacity as measured with non-trained tasks, implicating limited transfer. One common element in the training approach taken in all these studies is the use of computerized training and transfer tasks. The training programs involve rather extensive practice trials and demand the use of computer equipment and software. Moreover, the effects of this training (if found) may possibly only transfer to other computer-based tests and not to daily-life settings. In this framework, it is important to evaluate the potential of more ecologically valid, daily-life training techniques to improve response inhibition, both for theoretical and practical purposes (see hereafter). There are a number of previous studies exploring the effect of computer-based inhibition training programs on inhibition in daily-life contexts, such as those implicated in controlling food and alcohol consumption (e.g., Houben, 2011; Houben et al., 2011). However, examples of research employing the reversed design, using a daily-life inhibition training protocol to improve performance on computer-based inhibition tasks, are much scarcer (see Kida et al., 2005, for an example in the context of baseball playing). Of course, the ultimate goal of any training protocol is to improve the trainee’s performance on everyday (e.g., school) activities, not on some laboratory task. However, our study aimed to first assess, as a ‘proof of principle’, whether training in a daily-life setting can improve performance on well-controlled and validated computer-based inhibition tasks. Performance on such tasks may be easier to quantify than performance on everyday activities and has been shown to be predictive for everyday cognitive, social, and emotional functioning.

Besides potential practical benefits related to ease of integration in daily-life activities, one theoretical approach that motivated us to examine a daily-life inhibition training protocol is that of grounded or embodied cognition (e.g., Barsalou, 2008). This approach holds that action and cognition are inextricably linked. For example, human representations are multimodal and include bodily actions (e.g., Glenberg and Kaschak, 2002). Of primary importance for present purposes, bodily actions also contribute to the creation of mental representations (Boncoddo et al., 2010). For example, the execution of bodily actions, in the form of gestures during learning of a new concept, has been shown to have beneficial effects on retaining the acquired knowledge (Cook et al., 2008; Macedonia, 2014). Based on this theoretical approach and supporting evidence, we reasoned that inhibition training involving a game with different types of bodily actions might be particularly effective in yielding transfer effects. Furthermore, the required actions in such a game might be assumed to be more closely linked to the real world in comparison to the actions in most computerized tasks, which might further contribute to stronger transfer effects. Finally, as will be outlined below, the task structure of daily-life games might often be more complex than those involved in many standard computerized training tasks, possibly implicating a more efficient training protocol.

One potentially useful paradigm for real-life inhibition training in children is a game we call “Wesley says.” This game was first used by Strommen (1973) and later by Carlson (2005) in a battery of ecologically valid executive functioning tests for young children. The child is required to perform a specific action when the corresponding command to execute the action is preceded by the phrase “Wesley says,” but to refrain from making the response in the absence of this phrase. For example, if the game-leader says “Wesley says jump,” the child must jump; if the leader says “jump,” the child must not make any movements. This task has specific features that, theoretically, make it more demanding than is the case for a standard go/no-go task. In the “Wesley says” task the response prompt is presented as a verbal command, whereas it is a neutral visual stimulus in a standard go/no-go task. Also, the “Wesley says” task demands the execution or inhibition of a different motor response on each trial, whereas it is the same response on each trial in the go/no-go task. Finally, there are important differences in task structure. Specifically, the “Wesley says” game implicates a serial conditional or “feature-positive” discrimination procedure (e.g., Sambeth and Maes, 2006). Schematically, the participant must make a discrimination between X→A+^a^ and A–^a^ trials, with A representing the prompt to perform a specific action, X the verbal expression “Wesley says,” “+^a^” the execution of the prompted action and “–^a^” withholding the execution of the action. This implicates that the meaning of one and the same stimulus, in terms of response requirement, is conditional upon the presence or absence of a preceding stimulus. Instead, using this notation, a standard go/no-go task has a more simple structure, A+/B–, with A representing a go stimulus, B a no-go stimulus, “+” the execution of one simple motor response, and “–” no response. Previous animal and human research suggests that the former task structure (even if only one and the same action is required on X→A+ trials) implicates a more difficult-to- learn and/or -perform task, as expressed in lower response accuracy across trials during or after learning the relationships, than the latter task structure does (Dibbets et al., 2002a,b; Maes and Eling, 2007). All or some of these differences between the two types of task might implicate that training on the present task is more efficient and/or thorough than is the case for a training based on a standard go/no-go task.

In the present study, we used the “Wesley says” game to train response inhibition in 8- to 12-year-olds. The name of the game is based on a very popular domestic (Chinese) TV animation series for children called “Happy goat and gray wolf.” The animation was awarded the first prize in domestic animation by the National Broadcasting and TV Bureau. We used a standard computer-based visual go/no-go task to assess near-transfer effects. To test for transfer to another aspect of inhibition, interference control, we adopted the Stroop color–word interference task (MacLeod, 1991).

## MATERIALS AND METHODS

### PARTICIPANTS

All participants were in grades three to five in a primary school in Gansu province, China. Thirty-four children volunteered to participate. The participants had their primary caretakers sign informed consent forms. Participants were randomly assigned to either the training or control group according to the last two digits of the participant’s student number. Specifically, if the last two digits were odd, the child was assigned to the experimental group; if even, to the control group. A random assignment may be problematic in the context of training and transfer studies because this might result in unequal pre-training performance on the criterion tests (Green et al., 2014). However, this was not an issue in our study. If we would have created two groups that would have been perfectly matched on the pre-training measure of interest (e.g., the interference measures; see below), by first rank-ordering the values and then creating matched pairs, the mean, standard deviation (SD), and range of each of the two groups thus created would have been almost identical to that of our actual groups created by random assignment. Two participants in the training group failed to attend the post-training test because of a health problem; two participants in the control group refused to complete a particular task due to a misunderstanding of the experimental procedure. Hence, each group consisted of 15 participants. The mean age of the participants in the training group (10.07 years; SD = 1.28) did not differ from that in the control group (10.60 years; SD = 1.35; *F*(1,28) = 1.23, *p* = 0.28, η^2^ = 0.04). Both the training and control group consisted of seven boys and eight girls. Except for one boy in the training group, all children were right-handed. All participants had normal or corrected-to-normal vision and had no history of color blindness, neurological problems, or psychotherapy. The experimental protocol was approved by the Northwest Normal University Psychological Experiment Ethics Committee.

### MATERIAL

The pre- and post-training tests were programmed using E-prime 2.0. The stimuli were presented on a 14-inch display with a 1366 × 768 resolution and a 60-Hz vertical refresh frequency. Participants sat 60 cm from the monitor at a 3° angle.

### PRE- AND POST-TRAINING TESTS

#### Go/no-go task

A commonly used go/no-go task was employed to assess response inhibition prior to and after training. The task was conceptually identical to the tasks used in, for example, most of the studies on the effect of inhibition training referred to in the introduction. Across task versions, differing in probabilities of go and no-go stimuli, task execution has been shown to be associated with neurocognitive networks involved in motor inhibition (Rubia et al., 2001). The task consisted of one block of practice trials and four blocks of 100 experimental trials each. Each practice trial commenced with a white fixation point presented for 1000 ms in the center of a computer screen against a black background, followed by a 600-ms presentation of either the English letter X or Y. Thereafter, a blank screen was presented for 1000 ms. The next trial began immediately thereafter. The participant was instructed to respond to each X by pressing the letter “J” on a standard computer keyboard and to refrain from responding upon presentation of the letter Y. The participant went on with the experimental phase of the task after reaching an accuracy level of 85%. Each trial of the following two experimental blocks was identical to the trials in the practice phase. Each 100-trial block consisted of 70% trials with the letter X and 30% trials with the letter Y. In this way, a strong general response tendency was established to respond to the stimuli, which had to be suppressed on the relatively infrequent no-go trials. The order of trials was randomly determined. During the next two 100-trial blocks, the child had to press “J” to the letter Y and not respond to the letter X. In these blocks, the percentage of Y and X trials was 70 and 30, respectively. The child could have a break between trial blocks if desired and the total task lasted approximately 15 min.

#### Stroop color–word interference task

The Stroop color–word interference task was used to measure interference control during pre- and post-training sessions. The participants were required to indicate the color in which Chinese characters (Hanzi) or the symbols “####” were printed as quickly and accurately as possible by pressing either the letter F for the color red or the letter J for the color green on a standard keyboard. The corresponding keys were covered by a piece of red or green paper. The Hanzi characters represented the colors red or green and were printed in either red or green. The task comprised six trial types: two congruent trials, two incongruent trials, and two neutral trials. Congruent trials consisted of the Hanzi reflecting the word “red” printed in red and the word “green” printed in green. On the incongruent trials, the word “red” was printed in green and the word “green” was printed in red. Finally, on neutral trials, the symbols “####” were either printed in red or green. On each trial, a fixation cross was first presented for 500 ms, followed by a 1000-ms blank screen. Thereafter, the target stimulus (colored Hanzi or symbols) was presented for 1500 ms, followed by a blank screen that was presented for a variable duration between 600 and 1000 ms. The next trial started immediately thereafter. The task consisted of three blocks of 36 trials each, with breaks between blocks if desired by the child. Each block consisted of six presentations of each of the six trial types, for a total of six congruent, six incongruent, and six neutral trials. The order of trials was random and the session lasted about 15 min.

### TRAINING

The training game was played collectively on the school’s playground. The children stood in a circle or were lined up in several rows facing the experimenter. Before playing the actual training game, the experimenter instructed the children to perform a series of “silly” movements, such as touching the nose, stamping feet, or clapping hands. The children were then told that, in the following stage, the actual execution of the action depended on whether or not the command was preceded by the verbal expression “Wesley says.” If so, the child had to perform the corresponding action; if not, it had to stay still. The training began after the children had had the opportunity to practice, until they performed correctly on both trial types. The training consisted of 210 trials, presented in seven blocks of 30 trials each. Each block contained 15 trials with, and 15 trials without the “Wesley says” phrase. The order of trial types and the nature of the prompted actions were pseudo-randomly determined. About 1 s after the children completed their response, a new trial was presented. If the children refrained from responding to a “without Wesley says trial,” a new trial started after about 3 s. The experimenter gave feedback to individual children making a mistake.

### CONTROL MANIPULATION

The children in the control group played extracurricular activities that were unrelated to the training game, such as a game called “Throw the handkerchief,” or “Hawks catch chicken,” which are traditional child games in China. Like the training game, these games involve physical exercise. However, they do not contain a (clear) inhibition component.

### PROCEDURE

The experimenter who took part in the pre- and post-training tests sessions did not participate in the training session. The children were collectively tested during the pre- and post-training sessions in a quiet location. The children in the training group performed the daily 20-min “Wesley says” game on the school playground for 7 days, for a total of 140 training minutes. While the children were playing the training game, the children in the control group played the other extracurricular activities. The post-training Stroop task was performed one day after the last training game session; the post-training go/no-go task was performed on the next day.

### DATA ANALYSIS

The mean response time (RT) on go-trials and the percentage of trials with an error of commission (response on no-go trial) and an error of omission (no response on go trial) were recorded as outcome measures of the go/no-go task. Trials with an RT of less than 150 ms were excluded from the RT analyses. As summary measure specifically tapping response inhibition capacity, we subtracted the mean percentage of trials with a commission error (‘false alarms,’ FA) from the mean percentage of trials with a correct response on go-trials (‘hits’), with a large difference score reflecting a strong inhibitory capacity. For the Stroop task, we examined the mean RT and accuracy (percentage of trials with a correct response) for each trial type. Error trials and trials with a RT less than 200 ms were excluded prior to the RT analyses. As specific measures of interference control, we subtracted the mean RT on neutral trials from the mean RT on incongruent trials, and the mean accuracy score of incongruent trials from the mean accuracy score of neutral trials. In both cases, a low score represents strong interference control. The distribution of most of the measures was not normal and contained outliers. Therefore, we performed non-parametric analyses, analyzing the difference between groups with Mann–Whitney tests, and the difference between sessions with Wilcoxon tests. For all analyses, we report the corresponding *z*-score and used *p* < 0.05 as criterion for statistical significance. In addition to these main analyses, we performed exploratory analyses directed at assessing possible age and gender differences in the magnitude of transfer effects. These analyses were motivated by previous research on the development of different types of executive functioning, including inhibition. These studies suggest that there might be a continuing development from ages 8–12, the age range included in our study (Best and Miller, 2010). Moreover, as girls generally tend to outperform boys on executive functions (Carlson and Moses, 2001; Berlin and Bohlin, 2002), it might be that especially younger boys benefit more from training than older girls. As we only observed significant transfer for the go/no-go task (see below), we performed Pearson correlation analyses for each group separately, exploring the relationship between age and gender on one hand, and the post-pre difference score of the Hits-FA difference score (a high score reflecting a strong improvement from pre- to post-training assessment) on the other. For these analyses, we used the rank-ordered difference scores, because of a non-normal distribution of these data.

## RESULTS

### GO/NO-GO TASK PERFORMANCE

The top part of **Table 1** summarizes the score on the different measures of the go/no-go task, separately for each training condition and time of testing. During the post-training test, the children from the training condition made fewer errors of omission and commission, and had a larger inhibition difference score, compared to the pre-training test (all *z*s < –2.58, *p*s ≤ 0.01). None of the other differences between conditions and test sessions were significant (*z*s > 1.93, *p*s > 0.05).

**Table 1:** Groups’ mean (+SD) pre- and post training scores on the dependent measures of the go/no-go and Stroop tasks.

	Training group		Control group	
	Pre-training	Post-training	Pre-training	Post-training
**Go/NoGo**
GoRT	492.72 (73.72)	477.88 (58.33)	532.44 (141.15)	520.58 (122.24)
Omission Errors	**12.60** (7.66)	**8.67** (7.15)	10.87 (7.40)	11.67 (10.87)
Commission Errors	**13.27** (8.10)	**7.93** (7.51)	12.40 (9.10)	9.93 (16.16)
Hits – false alarms (FA)	**74.13** (15.42)	**83.40** (14.54)	76.73 (16.14)	78.40 (26.27)
**Stroop**
Incongruent response time (RT)	**757.42** (93.30)	*598.75 (91.66)	756.66 (132.45)	*718.51 (133.67)
Congruent RT	**690.53** (67.01)	*563.36 (66.56)	683.78 (99.36)	*692.62 (122.00)
Neutral RT	**732.46** (69.66)	*584.74 (77.59)	700.47 (116.12)	*690.10 (113.41)
Interference RT	24.95 (59.49)	14.00 (36.72)	56.19 (61.60)	28.41 (42.47)
Incongruent ACC	87.20 (19.06)	*93.80 (6.06)	91.73 (5.99)	*87.40 (12.26)
Congruent ACC	92.87 (6.49)	94.87 (4.31)	93.60 (5.44)	89.27 (13.39)
Neutral ACC	92.33 (6.72)	94.00 (6.85)	93.60 (5.43)	90.53 (12.72)
Interference ACC	5.13 (16.52)	0.20 (5.00)	1.87 (5.59)	3.13 (6.73)

Score for Errors represents percentage of trials with the error type indicated. RTs are in ms. Hits – FA is the difference between the percentage of trials with a correct response on Go trials and the percentage of errors on No-Go trials. ACC is accuracy, expressed as percentage of trials with a correct response. Interference RT and ACC represent the difference in, respectively, RT and accuracy on incongruent and neutral trials. For each row, values in bold significantly differ within subjects; the same holds for values of between-subjects comparisons marked with *.

### STROOP TASK PERFORMANCE

The lower part of **Table 1** displays the scores for the Stroop task. The children in the training condition performed significantly faster on incongruent, congruent, and neutral trials during the post-training test session than during the pre-training test session (*z*s < –3.29, *p*s = 0.001). Moreover, during the post-training session, the children in the training condition responded faster on all three trial types and had a higher accuracy score on incongruent trials than did the children in the control condition (*z*s < –2.37, *p*s < 0.02). None of the other differences between conditions and test sessions were significant (*z*s > –1.78, *p*s > 0.08).

### EXPLORATORY ANALYSES

For the training group, both age and gender were negatively associated with the go/no-go post-pre difference score (*r* = –0.40 for age and *r* = –0.77 for gender), implicating a greater training benefit for younger than older children, and for boys than girls. To compare, negative associations were also found for the control group, although these were much weaker (*r* = –0.24 for age and *r* = –0.33 for gender). However, probably also due to the small sample sizes, none of the correlations were significant (*p*s > 0.14), except for the training group’s correlation between gender and the difference score (*p* = 0.001). Using Fisher *r*-to-*z* transformation, we only found a tendency for the difference between the gender/difference score correlation to be more negative for the training group than the control group, *z* = –1.66, *p* = 0.097.

## DISCUSSION

This study assessed the effect of training response inhibition on two transfer tasks in 8- to 12-year-old children. For training, we used a game which was integrated into daily school activities, called “Wesley says,” and a double-blind randomized controlled experimental design. Results revealed that 7 days of playing the game significantly improved the children’s response inhibition ability, as measured with a non-trained computer-based go/no-go task. Specifically, relative to pre-training levels, there was a significant post-training decrease in both errors of omission and commission in the training group but not in the control group. Errors of omission are assumed to reflect symptoms of inattention, whereas errors of commission are held to be linked to inhibitory processes or impulsivity (e.g., Halperin et al., 1991). Hence, the observed decrease in both error types may reflect a combination of both enhanced attention and reduced impulsivity. Together, they implied a better inhibition capacity, as reflected in a significant increase for the training group in the difference between hits and false alarms from pre- to post-training session.

The results of the exploratory analyses suggest that the younger male children tended to show the strongest training benefit on go/no-go task performance. We also found reasonably strong (but considerably weaker than in the training group) corresponding negative correlations for the children in the control group, which likely reflect stronger simple test–retest benefits for younger than older children and for boys than girls. The larger benefits for younger than older children is in line with previous studies suggesting a continuing development of inhibitory capacities within the age range examined in the present sample. Accordingly, there might be more room for training-induced improvement in younger as opposed to older children. Likewise, for girls, generally tending to show superior executive functioning, there might be less room for improvement than for boys. However, these suggestions are all very preliminary and more research on age and gender differences in training benefits is clearly needed.

The training also affected performance on the second transfer task, a standard Stroop color–word interference task. Specifically, in the training group, but not the control group, the RTs significantly decreased from pre- to post-training test for all three trial types. Moreover, for each trial type, the children in the training group had shorter RTs than did the children in the control group, and the former children displayed a higher accuracy on incongruent trials than the latter. However, these changes and differences probably reflect more general effects on motivation or attention, rather than specific effects on inhibition capacity, as is also indicated by the absence of a significant training effect on the two interference measures.

The significant positive training effect on go/no-go inhibition performance in our study contrasts with the non-significant transfer effect reported by Johnstone et al. (2010). Specifically, in the latter study, training on a go/no-go task with pictorial stimuli did not have a significant effect on the performance on another go/no-go task with geometrical stimuli. Apart from the difference in the examined population (children with vs. without AD/HD), as outlined in the Introduction the training protocol used in our study differs in a number of respects from the (standard) go/no-go task used by Johnstone et al. (2010), which arguably makes the former task more difficult than the latter. This in turn might implicate that playing the present training game constitutes a more efficient and thorough inhibition training compared to repeatedly performing a go/no-go task. Moreover, the present effect can be considered a genuine transfer effect, given the difference between the trained and tested tasks in terms of task structure, stimulus modality, and response requirements. However, the most crucial aspect may have been the involvement of exercise or physical activity that, in the training group, was combined with motor inhibition. Interestingly, in a recent study, Jäger et al. (2014) found that 20 min of playful physical (sports) activities that contained cognitive engaging elements related to aspects of executive functioning (including inhibition) had an immediate positive effect on the performance on a computerized task measuring the capacity to ignore distracting information (interference control). However, this beneficial effect was no longer present 40 min after the sports period. Importantly, such short-term improvement was not seen in a control group that, like our control group, also received physical exercise but in the absence of a clear cognitive engagement. As in our study the go/no-go test was performed 2 days after the last training session, the results of our study suggest that repeatedly playing a game involving body activities that are specifically directed at motor inhibition might be sufficient for inducing longer-lasting beneficial effects, at least on other tasks tapping motor inhibition.

The absence of a significant training effect on the Stroop task regarding the critical inhibition-related interference measures suggests an important limitation to transfer (see also Thorell et al., 2009; Enge et al., 2014). Some authors argue that both the go/no-go and Stroop tasks measure inhibition of a pre-potent response and, therefore, tap the same underlying inhibitory process (e.g., Friedman and Miyake, 2004), with overlapping neural correlates (Aron et al., 2007). However, at least the nature of the to-be-inhibited response differs between the two tasks: a simple motor response in the case of the go/no-go task and a pre-potent reading response in case of the Stroop task. Moreover, the two tasks might measure different inhibitory processes, with (partly) different underlying neuronal systems (Nigg, 2000; Khng and Lee, 2014). For example, Nigg (2000) proposed that the Stroop task may be best conceptualized as a task measuring interference control, the ability to suppress attention for distracters that may potentially slow down the execution of the target response. The Stroop task involves the presentation of a two-dimensional stimulus, with the instruction to ignore one dimension and to respond to the other dimension. Hence, the structure of this task importantly differs from that implicated in both the “Wesley says” and go/no-go tasks. These differences may be at least partly responsible for the absence of positive transfer to the Stroop task. However, the training did have a more general effect on Stroop task performance, increasing response speed irrespective of trial type. This effect may reflect a general strategy shift (e.g., to give speed a higher priority than accuracy), but this did not result in a major change in inhibition capacity, as indexed by the interference measures.

### STUDY LIMITATIONS

Despite the preliminary evidence that the “Wesley says” training game has beneficial effects on a computerized non-trained go/no-go task, the present study has a number of limitations. First, the sample size was relatively small, thereby possibly preventing us from finding evidence for significant transfer of training effects to another task presumably tapping another aspect of inhibition. Second, unfortunately we were not able to conduct a follow-up study, to measure longer-term (i.e., >2 days) training effects. Third, we did not use an adaptive learning protocol, which may have resulted in stronger transfer effects. For example, it is feasible to gradually increase the difficulty of the task by creating additional, more complex conditional relationships after successful completion of more simple conditional relationships. For example, the X→A+^a^/A–^a^ discrimination task could be expanded to a X→A+^a^/Y→A+^â^/A– discrimination, in which the child is instructed to: (1) execute the prompted action (+^a^; e.g., “jump on right leg”) after the phrase “Wesley says,” (2) execute a “reversed action” (+^â^, e.g., “jump on left leg”) after the prompt to perform action “a” (jump on right leg) if the prompt is preceded by the phrase “Gray wolf says,” and (3) not respond to the prompt at all if there is no preceding phrase. Fourth, the study does not provide us with information concerning the mechanism underlying the transfer effect, neither at a cognitive/psychological nor neural level of description. For example, beneficial effects of our training task might specifically depend on the complexity of the task rule (e.g., Dowsett and Livesey, 2000). Concerning neural correlates, research directed at characterizing the neural changes evoked by inhibition training protocols are just beginning to emerge (e.g., Berkman et al., 2014), and more work on this issue is clearly needed. Fifth, the present study does not enable us to directly compare the merits of our (supposedly) ecologically more valid training game and those of a conceptually similar computer-based game. Finally, our research was restricted to a limited age-range and a non-clinical sample. Future research should explore the effects of the training game in other age groups and in clinical populations, such as in children with AD/HD.

## CONCLUSION

The present study provides preliminary, but promising, evidence of positive transfer effects of a novel training game to improve response inhibition capacities. One great advantage of the training game is its simplicity in terms of equipment requirements (none) and the ease with which it can be integrated into an everyday (school) context. Future research should replicate the positive transfer finding, while overcoming the limitations of the present study, such as creating better (adaptive) training conditions and directly comparing short- and long-term effects of computer-based and daily-life inhibition training protocols in different (age and clinical/non-clinical) populations.

## Conflict of Interest Statement

The authors declare that the research was conducted in the absence of any commercial or financial relationships that could be construed as a potential conflict of interest.
